# Costing oral cholera vaccine delivery using a generic oral cholera vaccine delivery planning and costing tool (CholTool)

**DOI:** 10.1080/21645515.2020.1747930

**Published:** 2020-06-12

**Authors:** Winthrop Morgan, Ann Levin, Raymond Cw Hutubessy, Vittal Mogasale

**Affiliations:** a Levin and Morgan LLC, Bethesda, MD, USA; b Initiative for Vaccine Research, World Health Organization, Geneva, Switzerland; c Policy and Economic Research Department, International Vaccine Institute, Seoul, South Korea

**Keywords:** Cholera, oral cholera vaccine, delivery cost, vaccination cost, campaign planning, program cost

## Abstract

Cholera is both an endemic and epidemic disease in many low and middle-income countries (LMICs). Strategies for cholera control include improving water, sanitation, and hygiene; providing early and effective treatment; and deploying oral cholera vaccine (OCV). This last strategy is relatively new, and countries considering its introduction are interested in knowing the potential cost not only of the vaccine, but also the cost of introduction. This paper describes the costing of OCV introduction in LMICs using a publicly available Excel-based tool known as the CholTool. It includes estimates of delivery cost categories which cover not only the service delivery costs (e.g. vaccine procurement, handling, storage, and transport; vaccination administration, monitoring supervision, and field support), but also the programmatic costs associated with introducing a new vaccine (i.e. microplanning, communication and training materials development, sensitization/social mobilization, and personnel training) to ensure that a comprehensive estimate is provided with health payer perspective. CholTool takes the user through a structured sequence of interlinked modules containing input parameter cells (assumptions), decision cells (variable selections), and formulas (calculations) to produce customized cost estimates based on standardized methods. The tool provides both financial and economic cost estimates, to ensure that both costs are available for consideration. Four examples of applications of CholTool are presented in three countries- one in Ethiopia, two in Malawi and one in Nepal. The estimates of economic delivery cost per dose (including service delivery and programmatic costs) were (in USD 2016): $2.89 in Ethiopia, $3.04 in Malawi1, $3.35 in Malawi2 and $3.06 in Nepal. A cost projection conducted before the campaign using the tool and a retrospective costing using the tool in Nepal resulted in no significant difference between economic delivery costs per dose.

## Introduction

The World Health Organization (WHO) recommends the use of Oral Cholera Vaccines (OCVs) along with water, sanitation and hygiene (WaSH) activities in both endemic and epidemic areas to prevent and respond to this extremely virulent, potentially lethal infection.^1,2^ Cholera vaccines are indicated in areas where water and sanitation is sub-optimal, outbreaks are likely to occur, and rapid treatment cannot be mobilized as the point of the vaccine is to prevent endemic cholera or outbreaks so that treatment is not required. OCVs offer demonstrated protection over 5 years with 65% efficacy.^3^

Immunization programs deliver OCVs through campaigns rather than routine immunization platforms. This is partially due to their non-traditional target populations; they not only target children under five but can also be used for all persons older than one year. OCV campaigns are usually conducted in high-risk areas, humanitarian emergencies, and settings with ongoing cholera outbreaks. Moreover, the use of OCVs is part of the comprehensive cholera elimination plan proposed by WHO and Global Task Force for Cholera Control (GTFCC) which includes effective coordination mechanisms, early detection and quick response to contain outbreaks and a targeted multi-sectoral approach to prevent cholera recurrence.^4^

Health programme managers considering the introduction of OCVs need to understand the potential costs to better plan for and secure financial resources. They not only need to estimate vaccine delivery costs as service delivery costs (vaccine procurement and vaccination administration), but also the programmatic costs associated with introducing a new vaccine (i.e. microplanning, communication and training materials development, sensitization/social mobilization, and personnel training). Furthermore, they need to compare alternative delivery scenarios to determine the optimal delivery strategy within their setting.

Estimating the procurement cost of the vaccine is straightforward. Two WHO pre-qualified OCVs Shanchol™ and Euvichol® were available at the time of the application of the CholTool in three countries (Euvichol-Plus® in a plastic tube replaced Euvichol® after 2016).The vaccine is labeled to be given in two doses to all ages older than one year. The vaccine is procured through the UNICEF procurement mechanism. The purchase price of OCV during the application of the CholTool in three countries (2015–2016) was 1 USD.85.^5^

Estimating delivery costs is less straightforward due to a lack of standardization in terminology and cost categories used in other studies. A systematic review of published and unpublished data from countries and organizations involved in OCV deployment in low- and middle-income countries (LMICs) reported vaccine delivery costs ranging (in 2014 USD) from 4 USD.75 to 6 USD.32.^6^ However, terms such as, “vaccine delivery costs “and “vaccine administration costs” were inconsistently defined; some referred to the cost of transporting the vaccine, while others referred to the entire cost of planning, procuring, preparing, and deploying the vaccine. The review noted that a lack of common methodology and standard categorization approach to reporting costs across organizations and geographical regions limited the robustness of their comparative analysis. Variation in delivery cost estimates and comparisons could benefit by having a standard methodology.

To address this limitation, Mogasale et al.^6^ recommended adapting the approach introduced by Hutubessy et al.^7^ for costing the delivery of Human Papilloma Virus vaccine to costing of OCV delivery. That is, they recommended that future OCV cost analyses use standard terminology to identify the types of activities included in the costings (for example, programmatic introduction activities; vaccine procurement; preparation for and conducting, supporting, and supervising of vaccine administration activities. They further recommended that cost analyses should have the following: 1) standardized cost categories (such as personnel, allowances, materials and supplies, equipment, and other direct costs) to facilitate comparative analysis 2) inclusion of both financial and economic costing methods in studies, and 3) presentation of capital and recurrent costs.

This study presents the CholTool, a user-friendly Excel -based model for calculating the “total delivery cost” of OCV delivery, which includes service delivery costs, and programmatic costs. It shows applications of using the CholTool to estimate OCV vaccine delivery costs in Ethiopia, Malawi and Nepal. The countries were chosen because they have either introduced OCV in communities where cholera is endemic (Ethiopia and Nepal) or used the vaccine after outbreaks have occurred (Malawi).

## Methods

The CholTool development team (Levin & Morgan LLC) created a conceptual model for costing OCV delivery based on economic methods used in the WHO Cervical Cancer Planning and Costing Tool.^7^ The CholTool is designed for costing from a payer/provider perspective (Appendix 1& 2). The tool user can make the perspective narrower or broader within this viewpoint. For example, the payer/provider may be defined narrowly as the Expanded Program on Immunization (EPI) department within a ministry, or it could be broadened to a government perspective. It can also include the perspective of an external partner such as the World Health Organization. The perspective is applied to determine which costs are included in the analysis (individual household costs are not included) and how included costs are considered as financial and economic (because donated resources are only included in economic costs).

The CholTool calculates both financial and economic costs. Financial costs are the value of resources to the buyer and include the value of actual resources purchased for the OCV campaigns such as allowances, supplies, transport and resources used in micro-planning, training, and sensitization/social mobilization. Financial costs include those monetary costs to the payer while economic costs include non-monetary costs such as donated goods such as vaccines and resources already purchased such as health personnel time.^8^ The user can choose which one is most appropriate depending on the objective of the analysis. For example, if they want to know additional costs incurred by the Ministry of Health (or other payer), they should focus on the financial cost calculation. An economic analysis is useful if the user is interested in evaluating the share of different sources of finance for the vaccine introduction. For example, they may want to know the share of total costs financed by the MoH, external partners, clients and the community. This analysis gives a more complete picture of resources that are tied up in the provision of the vaccine and their opportunity costs and should be used if a cost-effectiveness or cost-benefit analysis is to be conducted. The accompanying manual^8^ guides the user through financial and economic costing determination.

The tool used an ingredients-based methodology following the WHO-CHOICE (CHOosing Interventions that are Cost-Effective) project:” a “bottom up” or “ingredients based” approach, whereby each resource required for the intervention is identified and valued.”^9^ It is intended for use in incremental costing, which focuses on the additional costs associated with the introduction of a new intervention. The aim is to establish the impact of the change on costs relative to the situation prior to the change. Only additional costs directly incurred for OCV delivery preparation and implementation are included over and above existing infrastructures. An accompanying manual^8^ (Appendix 2), guides the user through the incremental costing method.

The CholTool is organized as a series of worksheets within a single workbook. It presents the user with a structured sequence of interlinked modules containing input parameter cells (such as the names of ingredients and their prices, the names of activities, and the names and numbers of target populations), decision cells (specifically, to choose a retrospective costing or a cost projection, and which currency – foreign or local – to apply in a given calculation area), and formulas (automatically calculating from inputs provided) to produce outputs. The tool can be run on any standard version of Excel 2010 or later, without enabling macros and has accompanying data collection forms. The intended user is a public health professional with training in health economics. A user manual accompanies the tool; it provides definitions of terms used, overviews of the navigation and methodology, and a step-by-step guide on how to collect and input data into the tool and interpret the results of the calculations made.

### Data requirements for the CholTool

The CholTool requires three main types of information: 1) basic demographic information; 2) vaccination strategy for the target population, and 3) types and numbers of unit resources (utilization) required for the vaccination and costs of each unit resource consumed (unit costs).

The demographic data required are the following: target population size, number of persons by service delivery strategy (fixed sites, mobile units or house-to-house visits), vaccine coverage assumption/achievement. The types and number of unit resources required include personnel, allowances, supply items required (such as stationery, IEC materials, forms, and cards), equipment related to cold chain, and other cost items relevant to the vaccination such as transport.

The prices of resources used in OCV campaigns are entered by the unit and multiplied by the quantity used. For example, if a day of a vaccinator’s time is required for a campaign, then the salary for one day of personnel time would be multiplied by one.

### Costing assumptions in the CholTool

Table 1 shows the activities and resources associated with each activity in the CholTool. The CholTool is arranged by the following activity groups:

- Programmatic: microplanning, IEC materials design and development, training, and sensitization/social mobilization
- Service delivery costs: vaccine procurement, vaccine administration (including program management), monitoring and supervision (including AEFI management, i.e. any untoward medical occurrence which follows immunization and which does not necessarily have a causal relationship with the usage of the vaccines, ^10^ and waste management.

**Table 1.** Vaccination Activities and Resource Requirements by Recurrent and Capital

Activity Group	Activity	Recurrent Costs	Capital Costs
Programmatic			
Programmatic	Micro-planning, Training, Sensitization, Social Mobilization	Health Personnel Time, Allowances, Supplies, Refreshments, Venue Rental, Equipment rental	
**Service Delivery**			
Vaccine Procurement	Shipping, Storage, Transport	Vaccines, Injection supplies, Freight, clearance, insurance and taxes, Transport and storage	Cold chain equipment, other
Vaccination administration	Vaccine administration, supervision, AEFI management, waste management, Other	Health Personnel Time, Allowances, Materials and Supplies, Fuel	Additional incinerators, Vehicles, Motorcycles, Boats, or Other etc.
Monitoring and Supervision	Supervision, Monitoring, AEFI Management	Personnel Time, Allowances, Transport	AEFI monitoring software/systems

* AEFI = Adverse events following immunizations, IEC = information education, communication

The user manual^8^ for the CholTool defines and explains each of these terms and their use in the CholTool.

Data entry into the costing tool involves a three-step process: 1) entering data on country characteristics, including administrative levels, names of sub-national areas, currencies and exchange rates, population and resource counts, and customized price lists; 2) listing the activities required for OCV introduction as well as data on resource unit costs; 3) specifying the number of times vaccination activities are conducted, e.g. number of trainings or micro-plannings.

Based on the inputs provided by the user, the CholTool computes an estimate of the financial and economic costs associated with procuring and delivering cholera vaccinations. These results are provided in local currency and US Dollars (or any other international currency desired, based on user-input exchange rates). Costs are reported by activity and subdivided into uniform cost inputs which span all activities, including: personnel, allowances, materials & supplies, equipment, and other direct costs. The Choltool calculates the cost per dose administered and the cost per fully immunized person and produces charts and graphs of the results. It allows the user to identify cost drivers for OCV campaigns.

For planning purposes, the user can identify and separate recurrent from capital costs (Table 1). Recurrent costs are the value of resources that last less than one year such as vaccines, personnel time, allowances and per diem, and transport. Most resources required for OCV campaigns are recurrent since these occur within one year. Capital costs, on the other hand, are resources that last longer than one year such as cold chain equipment. Capital costs (one-time) are amortized and discounted over the estimated useful life years (ULYs) of the investment. When calculating financial costs, straight line depreciation is used in the calculation of initial investment costs. That is, the cost of the item or activity s annualized through dividing it by the useful life years of the good or activity. For example, cold chain equipment could be expected to last for ten years and the equipment cost would be divided through by ten. A training program could be expected to last for five years and the total activity cost would be divided through by ten. Straight line depreciation assumes that initial investments are used up equally over the useful time period of the item. For economic costs, initial investment items are discounted as well as annualized. This type of depreciation assumes that people have time preference and prefer to use goods and services now rather than in the future. The CholTool allows the user to set the annual discount rate and the ULYs for each item. These are more fully defined and discussed in the CholTool User Manual.

### Applications of CholTool in LMICs

The CholTool was piloted four times in three countries: Ethiopia (2015), Malawi Campaign 1 (2015) and Campaign 2 (2016) and Nepal (2016).

### Preventive OCV campaign in Ethiopia

The Ministry of Health, in partnership with International Vaccine Institute (IVI), conducted an oral cholera vaccination campaign in 10 selected villages of Shashemenae, a rural district of Ethiopia with frequent outbreaks of cholera.^11^ They conducted two rounds of vaccination during four days each in February 2015 and March 2015, respectively. A total of 62,161 people were targeted, of which 47,137 people (76%) received the first dose and 40,707 (65%) received two doses.^11^

A vaccination implementation team was formed to conduct micro-planning at the national and facility levels with public health staff and community leaders. Programmatic activities included the following: 1) design of communications materials, 2) training of vaccinators and volunteers, and 3) sensitization of the community using sub-district community mobilization volunteers.

During the campaigns, 48 teams, each including a vaccinator, recorder, and crowd controller, conducted the vaccination activities. Monitoring for adverse events following immunization (AEFI) was conducted by vaccination teams and AEFI monitors located at health centers and hospitals.

### Outbreak response OCV campaign in Malawi

An OCV campaign was conducted in Nsanje District after a cholera outbreak (58 cholera cases and two deaths) occurred in response to flooding in March 2015. Some 70,000 displaced people (25.5% of the total population) were living in 19 camps. The total population eligible for OCV vaccinations, including the surrounding population, was approximately 160,000.^12^

The government of Malawi conducted the OCV campaign with support from IVI, John Snow, Inc. (JSI), and WHO. The following activities took place to prepare for the campaign: 1) development of materials to support training and social mobilization activities; 2) meetings with the District Executive Committee and local leaders to plan the campaign; 3) training of trainers, vaccinators (226 health workers), and volunteers; 4) social mobilization through local drama shows and community announcements. During the vaccination, 106 vaccination teams were formed, each with 2 vaccinators, 1 recorder and 1 crowd controller. In the first round, 156,592 (97.6%) of the target population were vaccinated over six days; in the second round, 108,237 (67.6%) received the second dose.^12^

A second OCV reactive campaign was conducted in February and March 2016 in three districts – Machinga, Phalombe, and Zomba.^11^ The campaign was supplied with doses of Shanchol from the International Coordinating Group (ICG) emergency stockpile using GAVI funding.^13^ The target population was 90,000, aged one and above, and 67,240 persons received both doses of OCV, with coverage of 58%. The vaccine was given at 98 vaccination posts, with 53 teams during the first round and 56 teams during the second. Also, 23 senior health surveillance assistants supervised the campaign.

### Preventive OCV campaign in Nepal

A two-round preventive OCV campaign was conducted in Nepal in November-December 2016. The population was distributed between one urban area – Ward no.5 of Nepalgunj; and a rural area – two villages, Sonpur and Udarapur of Nepalgunj district. The target population included about 30,000 people and the campaign achieved approximately 95% vaccination coverage for two doses.^14^ The vaccination was implemented through the routine immunization system functioning under MoH. The vaccination activities included sensitization at national and district levels, micro-planning, social mobilization and communication, media sensitization, training activities, vaccine administration, process monitoring, AEFI monitoring, and estimation of administrative vaccination coverage. Data collection for the CholTool began one week to one day before vaccination (cost projection) and two months after vaccination (retrospective or estimated with actual resource use). In the cost projection administration costs were projected based on the number of activities identified during the micro-planning, while in the retrospective costing, costs were collected as per actual expenditure reported.

### Data sources and other assumptions of CholTool

In Ethiopia, the costing team^11^ collected retrospective delivery costs with CholTool data collection forms at the Ethiopian Public Health Institute at Addis Ababa and the Primary Health Center at Shashemane two months following the 2015 OCV campaign. They interviewed program managers and coordinators involved in planning and implementing vaccination campaign.

In the first Malawi OCV costing study, the data collection team collected retrospective information on service delivery during the 2015 OCV campaign using CholTool data collection forms two months after the OCV campaign in Malawi for the first study from the payer organizations – i.e. the World Health Organization in Lilongwe, John Snow Int. and International Vaccine Institute in Seoul, South Korea. Data were collected at the national level.

For the second Malawi OCV study^13^ on the 2016 OCV campaign, the costing team collected retrospective data one year after the campaign using four structured questionnaires from CholTool. The team collected data from programmatic documents on microplanning and other activities as well as financial reports They also conducted interviews with program managers involved in implementing the campaign.

In Nepal, the costing team visited the country twice to collect data: one week before and two months after the vaccination campaign. In the cost projection, the team conducted interviews with the Program Manager, Coordinator and Finance Officers at the vaccination sites. After reviewing the micro-plan, key assumptions, projected quantities and prices were entered into the CholTool. During the retrospective estimation, the same people were interviewed to collect data on resource use in the OCV campaign activities after it had taken place. Also, actual expenditure records were reviewed.

## Results

Table 2 shows the administrative (based on number of doses administered) vaccine coverage rates for first and second doses in Ethiopia, Malawi Campaign 1 (Malawi C1), Malawi Campaign 2 (Malawi C2), and Nepal. The coverage rates for persons receiving both OCV doses for Ethiopia, Malawi C1, Malawi C2, and Nepal were 65%, 68%, 58%, and 80% respectively. The Malawi campaigns achieved higher administrative coverage for the first dose than the campaign in Ethiopia and Nepal, possibly because of their higher use of government health personnel to conduct the campaigns. The dropout between the first and second campaigns, though, was twice as high in Malawi than in Ethiopia and smallest in Nepal.

**Table 2.** Characteristics of Target Populations, Coverage, and Vaccine for Ethiopia and Malawi

	Ethiopia (2015)	%	Malawi Vaccination 1 (2015)	%	Malawi Vaccination 2 (2016)	%	Nepal (2016)	%
Target Population	62,161	100%	160,482	100%	90,000	100%	29,965	100%
Round 1	47137	76%	156,592	98%	108,483	121%	25,550	85%
Round 2 1^st^ dose* 2^nd^ dose	NA 40,707	NA 65%	26,599 108,237	18% 68%	NA 67,240	NA 58%	2,911 24,001	10% 80%
Dropout Rate	NA	14%	NA	31%	NA	38%	NA	5%
Vaccine Price per Dose	$1.85	NA	$1.85	NA	NA	NA	$1.85	NA

*Persons that received their first dose of OCV during the second round. Source: Teshome 2018, Msyamboza 2016 and Stop Cholera 2018,^8,9,11^

Table 3 shows the estimated financial and economic costs of conducting OCV campaigns by country. Total financial costs in 2016 USD varied considerably among the four campaigns and range from 143000 USD to 838,000, USD due to differences in target population size. Economic costs in 2016 USD similarly range from $ 160,312 USD – 895,000. USD Approximately 60%-70% of the total cost was for OCV vaccine procurement, followed by vaccine administration for Ethiopia and Malawi.

**Table 3.** Retrospectively estimated Financial (from donor Perspective) and Economic Costs of Conducting OCV Campaigns

	Ethiopia (US$20156		Malawi (US$2016) Vaccination 1		Malawi (US$2016) Vaccination 2			Nepal (US$ 2016)	
	LG electronics and IVI		Kia Motors, IVI and WHO		ICG and GAVI			Rotary club, IVI and others	
Sources of Financing	Financial costs	%	Financial costs	%	Financial costs	%		Financial costs	%
Programmatic									
Micro-planning	$14	0.01%	-	-	11,648	2.4%		$1,115	0.8%
Training	$2,482	1.0%	$13,292	1.6%	10,191	2.1%		$2,782	1.9%
Sensitization/ Social Mobilization	$7,264	3.0%	$24,336	2.9%	33,242	6.7%		$26,143	18.2%
Sub-Total Vaccine Program preparation	$9,760	4.0%	$37,628	4.5%	55,081	11.5%		$30,040	20.9%
Service Delivery									
Vaccination administration	$54,421	21.4%	$188,074	22.4%	76,238	15.9%		$18,341	12.8%
Monitoring and Supervision	$1,959	0.8%	-	NA	-	-		$930	0.7%
Vaccine Procurement	$180,873	73.8%	$613,082	73.1%	349,956	72.9%		$94,650	65.8%
Sub-Total Implementation	235,253	96.0%	$801,157	95.5%	$426,194	88.7%		$113,921	79,1%
Total	$245,013	100%	$838,785	100%	480,275	100%		$143,962	100%
Cost per dose	$2.77		$3.12		$2.74			$2.74	
Cost per FIP	$5.96		$7.68		$7.2			$6.0	
Non-vaccine cost per dose	$0.75		$0.84		$0.7			$1.0	
Non-vaccine cost per FIP	$1.1.60		$2.04		$2.0			$2.1	
	Ethiopia		Malawi					Nepal	
	Economic costs	%	Economic costs	%	Economic costs		%	Economic costs	%
Vaccine Program preparation									
Micro-planning	$125	0.05%	-	-	$78,649		13.4%	$1,453	0.9%
Training	$2,984	1.2%	$8,295	2.0%	$11,097		1.9%	$4,509	2.8%
Sensitization/ Social Mobilization	$8,718	3.4%	$24,336	2.7%	$38,889		6.6%	$29,993	18.7%
Sub-Total Vaccine Program Preparation Costs	$11,827	4.7%	$42,631	18.2%	$128,635		21.9%	$35,955	22.4%
Implementation									
Vaccination	$57,628	22.7%	$238,688	26.7%	$110,046		18.7%	$28,291	17.7%
Monitoring and Supervision	$3,633	1.4%	-	NA	-		-	$1,415	0.9%
Vaccine Procurement	$180,873	71.2%	$613,434	68.6%	$349,956		59.5%	$94,650	59.0%
Sub-Total Implementation	$242,134	95.3%	$852,122	95.2%	$460,002		76.7%	$124,356	77.6%
Total	$253,961	100%	$894,753	100%	$599,637		100%	$160,312	100%
Cost per dose	$2.88		$3.36		$3.35			$3.06	
Cost per FIP	$6.28		$8.16		$8.75			$6.68	
Non-vaccine cost per dose	$0.85		$1.08		$1.4			$1.3	
Non-vaccine cost per FIP	$1.81		$2.52		$3.5			$2.7	

ICG = International Coordinating Group; IVI = International Vaccine Institute; WHO = World Health Organization

While total costs of OCV vaccination varied due to differences in target population size and intensity of preparatory activities, the economic costs per dose in 2016 USD were similar – 2.70, USD 2.80, USD 3.40 USD and 3.20 USD – for Ethiopia, Malawi C1, Malawi C2, and Nepal, respectively. However, the cost per fully immunized person (FIP) was highest in Malawi C2 ($8.80) than in Malawi C1 ($6.80), Ethiopia ($5.90) and Nepal ($6.70) due to higher expenditure on micro-planning and sensitization activities (Table 3).

Cost projections and retrospective costings were completed in Nepal; the costs were compared (see Table 4) and found to be similar. In Nepal, the projected financial costs of the campaign were 142,227 USD while retrospective estimated costs were 143,963. USD Similarly, the projected economic costs were 165,403 USD while retrospective estimation showed it slightly lower at 160,312. USD

**Table 4.** Vaccine delivery cost (initial results) comparison when CholTool was used prospectively and retrospectively (US$ 2016)

Initial results (costs)	Prospective estimation		Retrospective estimation	
	Financial	Economic	Financial	Economic
Per dose administered USD	$1.0	$1.3	$1.0	$1.33
Per dose administered NPR	102	136	106	141
Per fully vaccinated person USD	$5.9	$6.8	$6.0	$6.7
Per fully vaccinated person NPR	597	694	639	711

NPR = Nepalese Rupee 2016; USD = United States Dollars 2016

**Figure 1.**
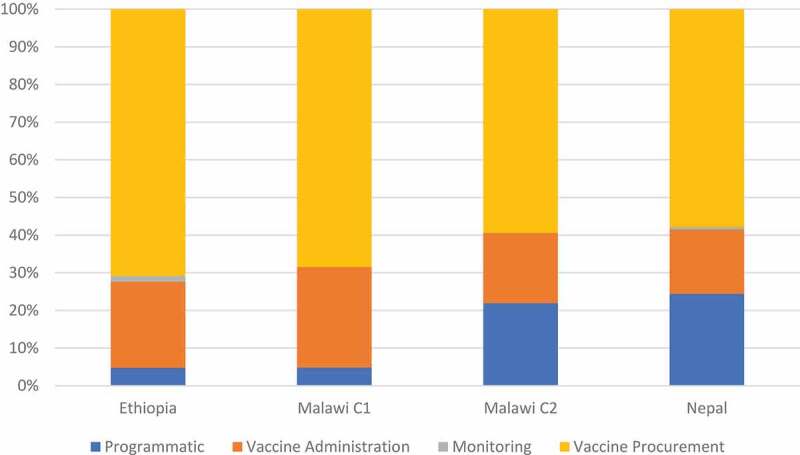
Economic Vaccine Delivery Cost Composition (%)

Vaccine delivery costs (non-vaccine) are shown in Figure 1. While vaccine administration is the cost driver for Ethiopia and Malawi C1, in Nepal, both sensitization/social mobilization and vaccine administration are the cost drivers and microplanning and vaccine administration for Malawi C2, respectively.

## Discussion

Countries are conducting OCV campaigns now that a low-cost vaccine has become available through Gavi funding. The OCV is used in preventive campaigns, emergency settings and as a response to outbreaks. When countries conduct vaccine campaigns, they must invest in critical programmatic activities, as well as service delivery costs to deliver the vaccines during the campaign.

Some of the differences in cost among the four studies were due to variation in OCV coverage rates, dropout rates, local price variation, and differing emphases on preparatory activities. OCV coverage was affected by the type of campaign – preventive or outbreak response – and intensity of microplanning, IEC/social mobilization and others. The higher dropout in Malawi C1 was due to the migration of flood victims back to their villages and dislike of the vaccine taste. The Nepal campaigns made extra efforts on sensitization, social mobilization and communication, as is evident by their higher costs under this category. This probably resulted in the lower dropout rate.

**Figure 2.**
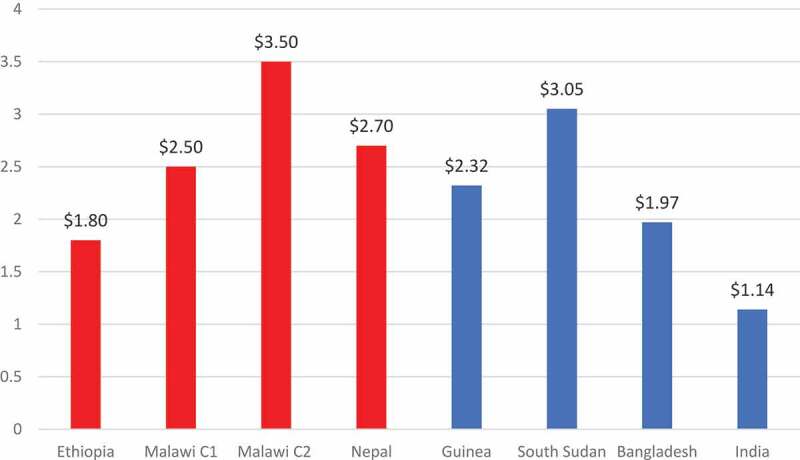
OCV Vaccine Delivery Cost per Fully Immunized Person (2016 USD)

The results from CholTool are similar to those of cost studies in other countries^14-18^ found in a recent review^6^ (see Figure 2). The OCV vaccine delivery costs (non-vaccine) per fully immunized person in Ethiopia, Malawi, and Nepal are similar to costs in Bangladesh and Guinea but greater than those in India and less than South Sudan. Cost differences can be attributed to variation in spending on introduction activities such as micro-planning.

In Nepal, the tool was used both for a cost projection before the campaign and retrospectively following the campaign, showing that it can be deployed both for planning purposes and also for input into economic evaluations. The experience suggests that the projected costs of a well-planned campaign executed as planned may not differ significantly from retrospectively estimated costs.

For estimating the costs of the four campaigns, the CholTool//was utilized by health economists. However, it can also be completed by health professionals with some health economics trained that have been learned to use the Excel user-friendly tool.

The CholTool can help countries to plan for conducting OCV campaigns since it calculates the total cost of a campaign and cost per dose and fully immunized person. It can be used to estimate the costs from a payer/provider perspective as in the case of the four OCV campaigns. Specifically, external agencies such as IVI, WHO, and UNICEF financed the majority of costs for these campaigns – training, IEC materials, vaccine procurement, personnel per diems, and fuel – while governments paid for health personnel salaries. It also can be used to estimate the resource requirements from a different perspective – the government perspective assuming that external financing will be phased out, to compare the cost of alternative service delivery strategies, and in estimating comparative cost-effectiveness of various strategies.

This study shows that the activites which drive the overall cost of introduction (other than vaccine procurement) include vaccine administration, sensitization/social mobilization and microplanning. Governments need to plan for these vaccine delivery costs so that they have adequate financial and human resources to ensure successful and sustainable programs.

## Limitations

The CholTool and its method of application have some limitations which should be noted. First, while there is guidance provided to the user in an accompanying user manual, completing the CholTool requires that the user understand costing in general and the structure and intended use of the tool in particular. It also is designed for estimating the cost over one year and not for medium-term planning.

## Conclusions

As outbreaks of cholera are occurring in many LMICs, governments need to plan to prevent future cases. As a part of comprehensive cholera elimination plan, governments in LMICs now have access to low-cost vaccine in their arsenal for reducing the incidence of cholera in addition to WaSH. This vaccine can be used preventively in areas with high-incidence of cholera or humanitarian emergencies and reactively in response to outbreaks. For governments to plan to introduce OCV effectively, they need cost estimates of OCV campaigns, particularly the non-vaccine costs needed for campaigns. The CholTool facilitates the task of planning and estimating the cost of OCV campaigns through helping immunization program planners and managers to calculate costs for the introduction of this vaccine through campaigns. The tool can assist countries to include introduction activities in their budget estimation as well as the cost of reaching different target populations. The cost data generated is also useful in understanding the value of money invested in a campaign based on systematic approaches such as cost-effectiveness analysis

## Supplementary Material

Supplemental Material

## Abbreviations

CholTool: oral cholera vaccine campaign costing tool
CEA: cost-effectiveness analysis
EPI: Expanded programme on immunization
FIP: fully immunized person
IEC: information, education, and communication
LMICs: low and middle-income countries
M&E: monitoring and evaluation
MOH: Ministry of Health
WHO: World Health Organization
