# Supplementary material for: Costing oral cholera vaccine delivery using a generic oral cholera vaccine delivery planning and costing tool (CholTool)
Source: Hum Vaccin Immunother. 2020 Jun 12;16(12):3111–8. doi: 10.1080/21645515.2020.1747930 (PMC8641596; doi:10.1080/21645515.2020.1747930)
Supplement: Supplemental Material [file KHVI_A_1747930_SM8430.zip › Appendix1. CHOLTOOL User Manual_October2015_final.pdf]

CHOLTOOL User Manual

Version 1.0

This guide and tool was developed by Ann Levin and Winthrop Morgan under the supervision of Vittal Mogasale of the International Vaccine Institute and Raymond Hutubessy of the World Health Organization.

The work was made possible by the generous support of the International Vaccine Institute.

Published [ ].

Recommended Citation: IVI and WHO. CHOLTOOL: Planning and Costing User's Guide, Seoul, South Korea.

Contact information:

Table of Contents

Introduction to Cholera 5
What the CHOLTOOL Does 6
Cost Components of Oral Cholera Vaccination 7
Recurrent Costs 7
Capital Costs: Introduction Costs, Supplemental Cold Chain and Other Equipment 7
Financial and Economic Costs 8
The CHOLTOOL Structure 11
Color Coding in CHOLTOOL 11
Software Requirements for CHOLTOOL 13
Navigating theCHOLTOOL 13
Gathering and Entering Data for CHOLTOOL 14
Cover Page 14
STEP 1: Setup the Tool for Use in a Specific Context 15
A. Complete the Time Series Worksheet 15
B. Complete the Customization Worksheet 15
C. Complete the Counts Worksheet 15
D. Complete the Currency Rates Worksheet 16
E. Complete the Resources Sheet 16
STEP 2: Estimate the Cost of Each Unique Single Activity 17
A. Estimate Vaccine Procurement and Shipping Activity Costs 17
B. Estimate Microplanning Activity Costs 18
C. Estimate Communication Material Development and Production Activity Costs 19
D. Estimate Training Activity Costs 19
Estimate Pre-Campaign Sensitization Activity Costs 20
E. 20
F. Estimate Social Mobilization Activity Costs 20
G. Estimate Vaccination Site Deployment Costs 21
STEP 3: ESTIMATE NUMBERS OF EACH ACTIVITY 22
A. Estimate the Number of Each Vaccine Procurement and Delivery Activity Needed 22
B. Estimate the Number of Each Microplanning Activity Needed 22

C. Estimate the Number of Each Sensitization Activity Needed 23
D. Estimate the Number of Each Sensitization Activity Needed 23
E. Estimate the Number of Each Training Activity Needed 23
F. Estimate the Number of Each Social Mobilization Activity Needed..............................23
G. Estimate the Number of Each Activity Needed During Round 1 of Vaccination........23
H. Estimate the Number of Each Activity Needed During Round 2 of Vaccination........23
STEP 4: REVIEW RESULTS AND ANALYSIS 23
A. Review the Cost Summary...23
B. Review the Analysis...23
Presentation of Results 25

Introduction to Cholera

Cholera is an acute, rapidly dehydrating diarrheal disease transmitted through water or food contaminated with the bacterium, Vibrio cholera O1 (or less frequently, O139), primarily in areas with poor access to safe drinking water and adequate sanitation. Rapid dehydration can lead to death within 24 hours in up to 50% of cases if not treated with intravenous or (for less severe cases) oral rehydration. With proper treatment, the case fatality rate may be reduced to much less than 1%. However, the poor and marginalized populations at greatest risk of cholera often lack ready access to adequate health care facilities, and the use of oral rehydration therapy (ORT) with diarrhea is inadequate and declining in many cholera-affected countries.

Cholera occurs both as endemic disease and in outbreaks, which can include large, explosive epidemics. Since the late 1990s, cholera epidemics have appeared in growing frequency, size and duration in Africa – including the 2008/09 epidemic in Zimbabwe, in Asia, and most recently in Haiti. Many outbreaks in the past decade have lasted up to a year or longer and are characterized by case fatality rates of 4% of higher. The severity of some of these outbreaks, including the Haiti epidemic, may be linked to the emergence of new, more virulent hybrid strains of V. cholerae O1 El Tor that produce the classical cholera toxin.

The potential for controlling cholera with the use of oral vaccines in cholera-affected countries increased since a new, lower cost vaccine (Shanchol™) was developed specifically for use in endemic countries and pre-qualified by WHO in 2011. While also a killed, two-dose vaccine, it has double the antigen (LPS) content of Dukoral®, contains both V. cholerae O1 and O139 strains, and lacks the cholera toxin component – thereby not requiring administration with a buffer or water. It has also been shown in a clinical trial in Kolkata, India to provide sustained protection over at least three years in all age groups (66% overall) [Sur et al. 2001]. The advent of this new vaccine, along with growing concerns about the continual incidence of the disease and potential for large outbreaks, as well as the emergence of new, more virulent strains of V. cholerae [Siddique 2009] led WHO in 2010 to recommend that cholera control, including the use of oral cholera vaccine (OCV), "be a priority in endemic areas", and that pre-emptive and possibly reactive vaccination be considered to prevent or halt outbreaks [WER 2010].

In order to facilitate decision-making on these interventions, program managers and policymakers need information on the projected costs of introducing OCVs through campaigns in endemic countries or as reactive vaccination to prevent or halt outbreaks.   The CHOLTOOL has been developed to assist governments to estimate both retrospective and prospective costs of OCV campaigns and is described in detail in this user guide.

What the CHOLTOOL Does

The costing tool enables the user to estimate the value of incremental (additional) resources required to add oral cholera vaccination campaigns to an existing immunization programme. That is, it only estimates the value of new resources needed and does not include the cost of other goods and services (e.g. transport) already being used for other vaccines. For example, it does not estimate the cost of transporting oral cholera vaccine (OCV) if this is part of the same transport used to deliver other vaccines from the central warehouse to the periphery in the country.

The quantity of resources required to introduce OCV to national immunization programs (NIPs) will differ from other vaccines since it targets all persons over the age of one year rather than infants, pregnant women, or adolescent girls. The coverage for this vaccine will depend on the percentage of the population that visit the outreach sites and are vaccinated with OCVs. In order for the campaign coverage to be high, it is important to conduct sensitization and social mobilization to inform the population of the benefits of getting the vaccine as well as the times and locations that it will be offered. The CHOLTOOL enables the user to estimate the additional resource requirements based on the specific strategy that will be used for the country.

The CHOLtool provides estimates of two cost measures: 1) total costs of adding the OCV to specific areas; and 2) cost per fully immunized person. It differentiates **recurrent** (operational**)** and **capital** costs as well as **financial** and **economic** costs. It also present expenditures required for initial investments required for the OCV vaccine introduction.

Cost Components of Oral Cholera Vaccination

The CHOLTOOL allows the user to estimate the costs of activities that take place during the introduction of OCV vaccination into a national immunization program. These activities include the following: vaccines and injection supplies (includes procurement, transport and storage), micro-planning, training, social mobilization and IEC, service delivery of vaccines to target populations, supervision and monitoring (includes AEFI management), and waste management.

In the following section, the differences between types of costs are discussed.

Recurrent Costs

Recurrent costs are the value of resources that last less than one year. These include program costs such as the value of personnel time, transport, maintenance, monitoring and evaluation, and supervision.

Table 1. Vaccination Activities and associated Recurrent Costs

Vaccination Activity	Recurrent Costs
**Vaccine Procurement, Transport and Storage**	Vaccines, Injection supplies, Freight, clearance, insurance and taxes, Transport and storage
**Micro-planning for Campaigns**	Health Personnel Time, Allowances, Supplies, Refreshments, Venue Rental
**Sensitization/Social Mobilization**	Health Personnel Time, Allowances, Supplies, Rental of Equipment, Refreshments, Venue Rental
**Short-term Training for Campaigns**	Health Personnel Time, Allowances, Supplies, Refreshments, Venue Rental
**Vaccination (includes Vaccination Site, Command Center, Mop-up Costing)**	Health Personnel Time, Allowances, Supplies
**Supervision and Monitoring**	Supervisor Time, Driver Time, Allowances, Transport, Tally Sheets, AEFI Surveillance
**Waste Management**	Fuel for Glass Disposal

Capital Costs: Introduction Costs, Supplemental Cold Chain and Other Equipment

Capital costs are the value of resources that last longer than one year such as cold chain equipment and vehicles.   Since many OCV campaigns are a onetime intervention and make use of existing resources, few capital goods and services are purchased.   If OCV preventive

campaigns were to be introduced on an annual basis or every five years in cholera endemic countries, some capital goods and services will be purchased. For example, initial training curriculum and IEC materials may be developed and some equipment could be purchased (see Table 2).

Calculation of capital costs differs from recurrent ones since these are annualized and/or discounted depending on the purpose of the analysis and whether financial or economic costs are preferred.

Table 2. Vaccination Activities and associated Capital Costs

Vaccination Activity	Costs
**Vaccine Procurement and Storage**	Additional Cold chain Equipment Requirements
**Training**	Initial Training, Curriculum Development,
**Sensitization/Social Mobilization**	IEC Material Development, Sensitization Meetings
**Waste Management**	Additional incinerators or other equipment for disposal of glass vials
**Other Costs**	Additional Vehicles, Motorcycles, Boats, Bicycles, etc.

Financial and Economic Costs

The CHOLTOOL calculates both financial and economic costs. The user can choose which one is most appropriate depending on the objective of the analysis. If they want to know the additional costs incurred by the Ministry of Health, for example, they should focus on the financial cost calculation.

Financial costs are the value of resources to the buyer and include the value of actual resources purchased for the OCV campaigns such as allowances, supplies, transport and resources used in micro-planning, training, and sensitization/social mobilization.

Economic costs comprise the value of all outlays for the vaccine introduction as well as those already paid for by the Ministry of Health and other sources of financing, e.g. the salaries of health personnel, vaccines paid for by partners, and time of volunteers. This analysis is useful if the user is interested in evaluating the share of different sources of finance for the vaccine introduction. For example, they may want to know the share of total costs financed by the MoH, external partners, clients and the community. This analysis gives a more complete picture of resources that are tied up in the provision of the vaccine and their opportunity costs and should be used if a cost-effectiveness or cost-benefit analysis is to be conducted.

Capital costs are calculated differently depending on whether financial or economic costs are being estimated. When calculating financial costs, straight line depreciation is used in the calculation of capital costs. That is, the cost of the item is annualized through dividing it by the useful life years of the good. For example, cold chain equipment could be expected to last for ten years and the total cost would be divided through by ten. Straight line depreciation assumes that capital goods are used up equally over the useful time period of the item. For economic costs, capital goods are discounted as well as annualized. This type of depreciation assumes that people have time preference and prefer to use goods and services now rather than in the future.

Table 3. Resources by Vaccination Activity for Financial and Economic Costs

Vaccination Activity	Financial Costs	Economic Costs
**Procurement of Vaccines and injection supplies**	Cost of vaccines and injection supplies to government<br>Cost of freight, clearance, insurance and taxes<br>Storage and transport	Cost of vaccines and injection supplies regardless of source of financing<br>Cost of freight, clearance, insurance and taxes<br>Storage and transport
**Micro-planning**	Per diems and travel allowances<br>Venue rental<br>Transport	Personnel time spent in meetings<br>Per diems and travel allowances<br>Venue rental<br>Transport
**Training**	Development of training materials<br>Per diems and travel allowances<br>Venue rental<br>Transport<br>Training Materials<br>Stationery	Value of personnel time spent on training<br>Development of training materials<br>Per diems and travel allowances<br>Venue rental<br>Transport<br>Training Materials<br>Stationery
**Sensitization/Social Mobilization**	Facilitator time in meetings<br>Per diems and travel allowances<br>Stationery<br>Printing of materials<br>Production of TV and/or radio spots	Value of personnel, and volunteer time spent on material development and other activities<br>Facilitator time in meetings<br>Per diems and travel allowances<br>Stationery<br>Printing of posters and leaflets<br>Production of TV and/or radio spots
**Vaccination**	Transport fuel<br>Personnel allowances to travel to vaccination sites<br>Supplies	Value of personnel time spent on vaccination<br>Transport fuel<br>Personnel allowances to travel to vaccination sites<br>Supplies
**Supervision and Monitoring**	Tally sheets or registers<br>Pens and pencils	Value of personnel time spent on supervision

Vaccination Activity	Financial Costs	Economic Costs
	Vaccination cards<br>Materials for surveillance	Tally sheets or registers<br>Pens and pencils<br>Vaccination cards<br>Materials for surveillance
**Waste Management**	Purchase of incinerators (annualized)<br>Fuel<br>Transport	Purchase of incinerators or other disposal for glass vials<br>Transport
**Cold chain equipment**	Cold chain equipment (annualized)	Cold chain equipment (annualized and discounted)

Table 3 presents a comparison of resources included in cost estimation based on whether financial or economic costs are being calculated. For micro-planning, for example, the value of personnel time spent in meetings is included in economic costs but not in financial costs.

The main differences between financial and economic costing are threefold:

1) The time spent by health personnel and volunteers is valued in economic costing since there is an opportunity cost to this time – i.e. the workers are unable to spend time on other activities when they are occupied with OCV vaccination - but are not included in financial costs since these are already paid for with government salaries
2) The value of donated goods and services is included in economic costs but not in financial costs since there is an opportunity cost to their use; and
3) Capital costs are calculated differently for financial and economist costs.

A glossary of terms used in the cost analyses in the CHOLTOOL is in Appendix 1.

The CHOLTOOL Structure

The CHOLTOOL has five sections:

1) TOOL SET UP
2) SINGLE ACTIVITY COSTING
3) ESTIMATE NUMBERS OF EACH ACTIVITY
4) RESULTS AND ANALYSIS, and
5) APPENDIX.

A link at the top of each page takes the user to the Table of Contents. The table of contents entitled ‘CONTENT’ allows the user to go to any section by clicking on the “Go to Table of Contents” hyperlink at the top left of each page.

The following is a description of the five sections:

1) The TOOL SET UP section includes the following worksheets: TIME SERIES, CUSTOMIZATION, COUNTS, CURRENCY RATES, and RESOURCE LIST.
2) The SINGLE ACTIVITY COSTING section has the following worksheets: VACCINE, MICROPLANNING ACTIVITY COSTING, COMMUNICATION MATERIALS, TRAINING ACTIVITY COSTING, SENSITISATION ACTIVITY COSTING, SOCIAL MOBILIZATION ACTIVITY COSTING, VACCINATION ACTIVITY COSTING – ROUND 1, and VACCINATION ACTIVITY COSTING – ROUND 2. These worksheets will be described in detail in the section on Data Entry.
3) The ESTIMATE NUMBERS OF EACH ACTIVITY section has the following worksheets: VACCINE PROCUREMENT AND DELIVERY, MICROPLANNING, SENSITIZATION, COMMUNICATION MATERIALS, TRAINING, SOCIAL MOBILIZATION, SERVICE DELIVERY (ROUND 1 – VACCINATION), and ROUND 2 – VACCINATION.
4) The RESULTS AND ANALYSIS section has the following worksheets: COST SUMMARY and ANALYSIS.
5) The APPENDIX section has one worksheet – CHECKS.

Color Coding in CHOLTOOL

In the CHOLTOOL, cells are color coded and shaded to indicate their purpose. That is, the color, shading, or border indicates whether these are for 1) inputting data, 2) linked to another cell in the workbook, 3) calculated, 4) not filled, or 5) for labels, as can be seen in Table 4.

Table 4. Color Coding in CHOLTool

Cell	Font Color	Cell Background Shading	Cell Border	Symbol
**Input**	Blue	Yellow	Solid line	None
**Dropdown**	Black	White	Solid Line	None
**Linked Cell**	Orange	White	Double orange line	None
**Check Cell**	White	Grey	Double line	None
**Note**	Black	Yellow	Solid line	
**Calculated Number**	Black	White	Solid line	None
**Total**	Black	White	Solid Line	Bold
**Hyperlink**	Magenta	None	None	None
**Warning Text**	Red	White	None	None
**Explanatory**	Grey	White	None	Italics
**Heading**	Black	White	None	None
**Title**	Blue	None	None	Bold

Figure 1 shows an example of color coding in the MICRO for vaccination.

Figure 1. Example of Color Coding used in the MICRO Worksheet

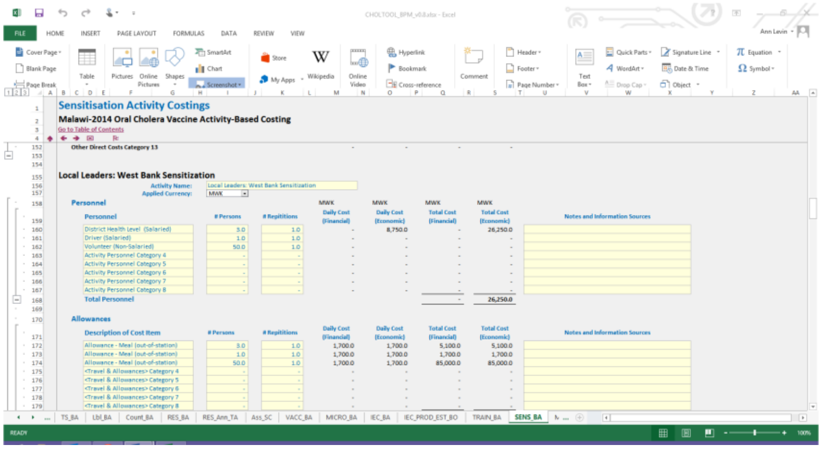

Software Requirements for CHOLTOOL

The CHOLTOOL is designed to be used with Microsoft Excel 2010 so that it can make use of the dropdown menu feature. It can also be used with Microsoft Excel 2007 but the user must fill in some information manually in a few places where there are dropdown menus.

Navigating theCHOLTOOL

The Table of Contents page allows the user to see what is contained in the tool and navigate to each content area.

Figure 3 shows a screenshot of the ‘CONTENT’ worksheet. It shows the sections as well as the name of worksheets within each section. The user can click on any of these to go directly to any of the worksheets.

Figure 2. Screenshot of CONTENT Worksheet

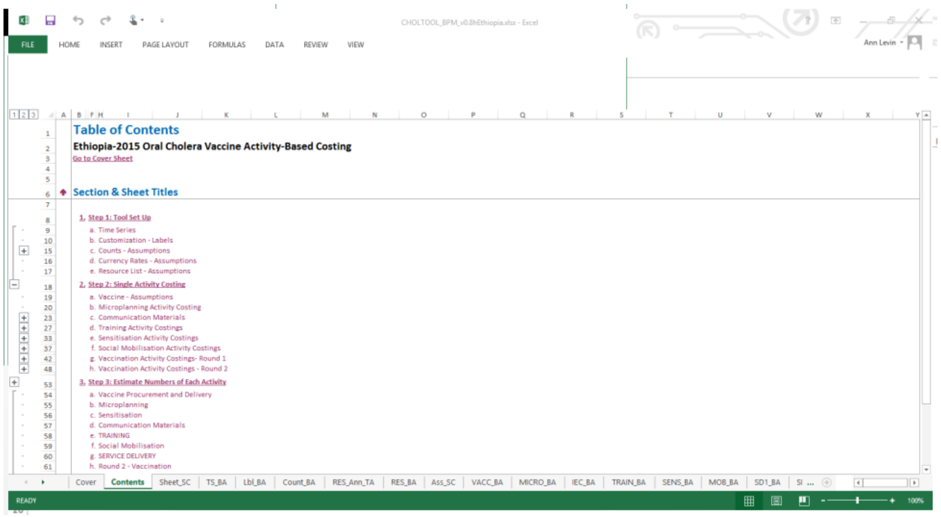

Gathering and Entering Data for CHOLTOOL

Before entering data, the user should examine the worksheets under SETUP and SINGLE ACTIVITY COSTING and determine whether there is consensus on the service delivery strategy to be used - i.e. when and where will the vaccination take place?; What is the plan for training? What other assumptions are required for the OCV vaccination to take place in their country?; Having information on the service delivery strategy is a necessary input into the tool.

After the strategy has been defined, the tool can be used to estimate/project the costs of implementing one strategy or comparing the costs of implementing two or more strategies. For example, the government may want to compare the costs of having ten vaccination sites vs. twenty vaccination sites. In that case, the user should make a copy of the CHOLTOOL and fill in the costs for each of the strategies so that these can be compared.

After the user has identified the strategies to be costed in the CHOLTOOL, s/he should gather together the data required for the analysis. A list of data that needs to be collected for the tool is given in Appendix 2. Once the user has gathered the data needed for the CHOLTOOL, he or she can begin the data entry.

Cover Page

The cover page worksheet is entitled COVER and a screenshot is shown in Figure 2.

Figure 3. Screenshot of Cover Sheet

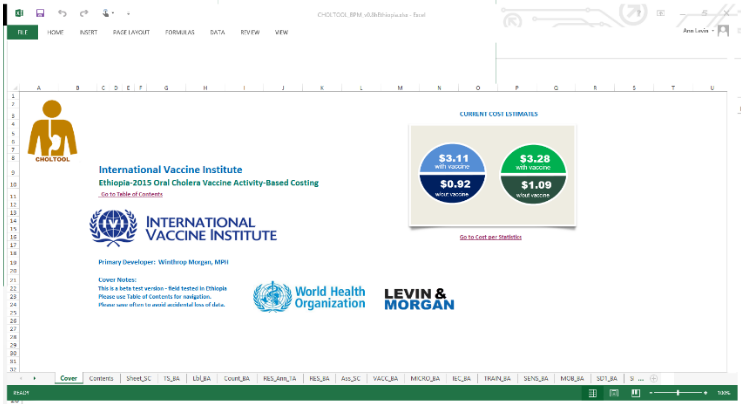

STEP 1: Setup the Tool for Use in a Specific Context

As mentioned above, the SETUP worksheets are entitled TIME SERIES, CUSTOMIZATION, COUNTS, CURRENCY RATES, and RESOURCE LIST.

A. Complete the Time Series Worksheet

In this worksheet, the user should fill in the financial year and year end for the OCV vaccination.

B. Complete the Customization Worksheet

The CUSTOMIZATION worksheet (see Figure 4) is used to enter information on country characteristics. First, the user should enter the name of the country. Once this information is entered, then the country name automatically is found on the other worksheets in the workbook.

Next, the user should fill in the names of sub-national level name labels such as national or central, second level areas such as districts, and third level areas such as health facilities.

Next, the user should enter the facility types for support facilities and field vaccination sites.

Finally, the user should enter the target population. In most cases, this will be persons older than 1 year of age.

Figure 4. Screenshot of Customization Worksheet

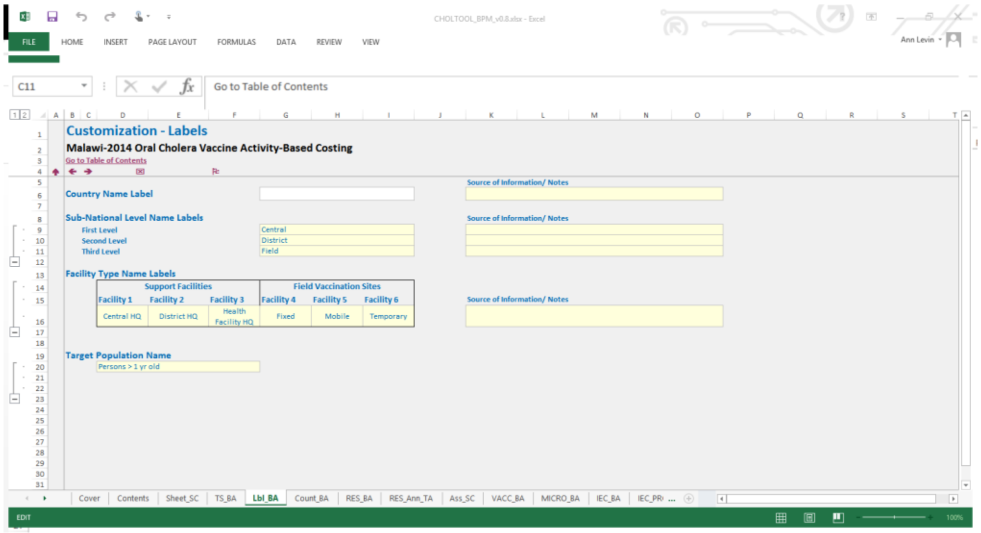

C. Complete the Counts Worksheet

This worksheet is used to enter information for each vaccination site.

In the first column, the user should fill in the names of the vaccination areas. The user can fill up to fifty names of these areas. If s/he wants to fill in more than fifty names, then they should

call the technical assistance number for the CHOLTOOL. (Cells are locked) In the second column, fill in the target population for each vaccination area.

In the third column, list the number of fixed sites in each vaccination area. In the fourth column, list the number of mobile sites in each vaccination area. In the fifth column, list the number of temporary sites in each vaccination area. In the sixth, seventh and eighth columns, list the number of vaccinators, volunteers, and local leaders by vaccination site.

D. Complete the Currency Rates Worksheet

In this worksheet (see Figure 5), the user should enter the currency code and exchange rates (exchange rates with the US$ or other foreign currency). This information can be found on the Ministry of Finance website or at other economic websites. In the second table, enter the annual inflation rate and discount rate.

Figure 5. Screenshot of the Currency Rates Worksheet

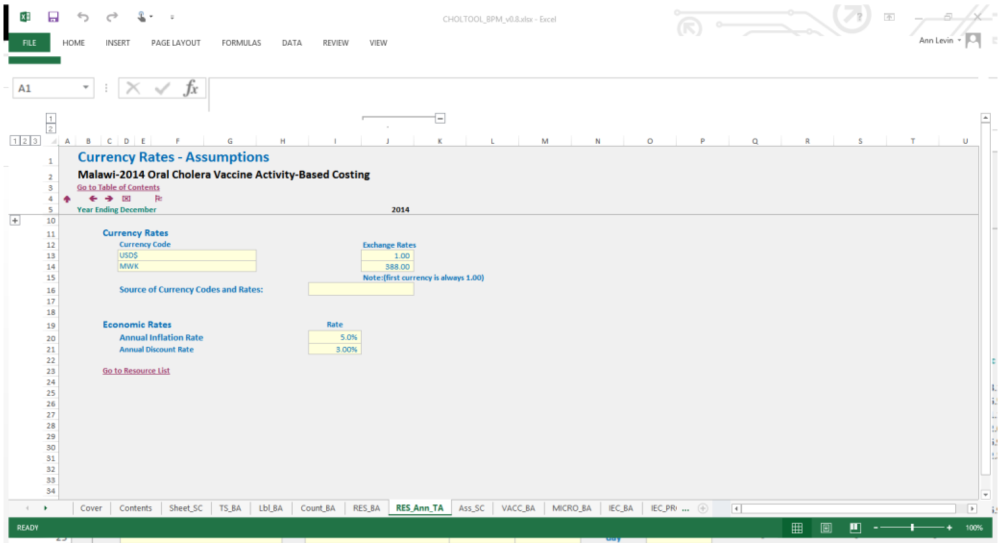

E. Complete the Resources Sheet

The RESOURCES worksheet (see Figure 6) is where data on cost per unit for resources used in OCV campaigns are entered. In the first table, enter in the types of health staff that work in OCV campaigns as well as their daily salary. Since most of these health staff are government employees, no financial cost will be incurred for salaries. However, the value of the health staff time will be shown under economic costs since there is an opportunity cost for these workers.

In the second table, enter the amount of allowances by type of health staff.   In the fourth and fifth tables, enter in the price per unit for supplies and equipment, respectively. In the sixth table, enter in the price per unit for other costs.

Figure 6. Screenshot of the Resources Worksheet

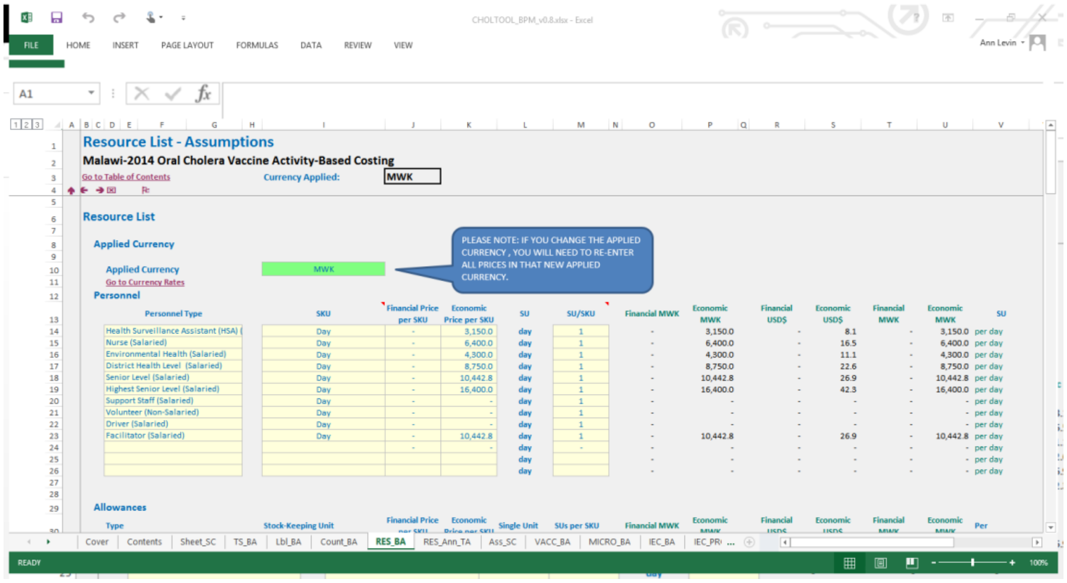

STEP 2: Estimate the Cost of Each Unique Single Activity

The second section is for SINGLE ACTIVITY COSTING. In these worksheets, users should enter assumptions for estimating costs of each activity.

A. Estimate Vaccine Procurement and Shipping Activity Costs

In this worksheet, data on the vaccine, its coverage and transport and storage are entered (see Figure 7). In the first table, fill in the predicted coverage for Rounds 1 and 2 are given as well as the dropout rate between Rounds 1 and 2 if the data are prospective. Otherwise, add in actual coverage if the data are retrospective.

In the second table, enter in the wastage rate and buffer/reserve stock. In the third table, enter in the number of vaccines for administration. In the fourth and fifth, fill in the price per dose of OCV and vaccine add-on charges, respectively. Vaccine add-on charges include insurance, wharfage and handling as well as transport to stock management centre and/or central warehouses.

Table 7. Screenshot of Vaccination Worksheet

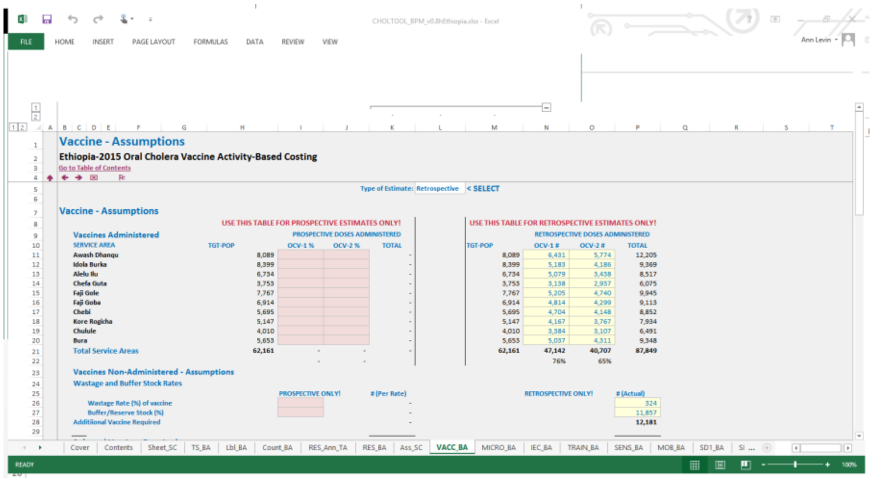

B. Estimate Microplanning Activity Costs

In this worksheet (see Figure 8), information on resource use for planning meetings at the national, district and vaccination area levels are entered.

The user should enter assumptions for estimating the cost of micro-planning: 1) the health personnel that attend planning meetings and the number of hours that they spend or spent in meetings; 2) number and frequency of allowances paid to health personnel in planning meetings; 3) number and frequency of supplies needed for micro-planning; 4) equipment used in micro-planning; and 5) other costs used in micro-planning.

Figure 8. Screenshot of Micro-planning Worksheet

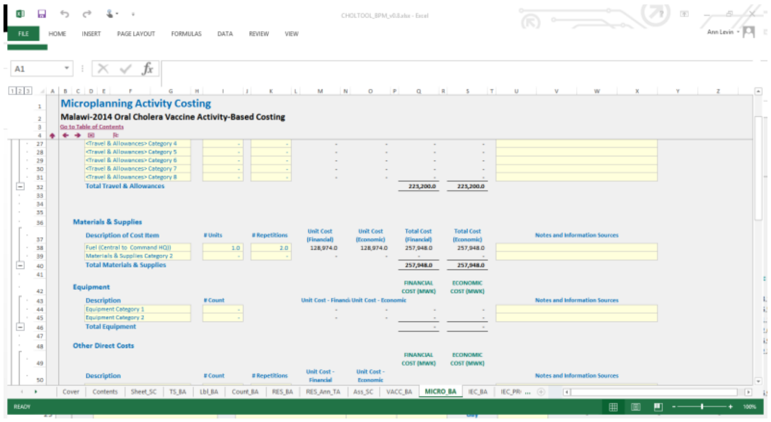

C. Estimate Communication Material Development and Production Activity Costs

In this worksheet (see Figure 9), the user should enter the assumptions for estimating the cost of activities on communication material development. Fill in the quantities of resources used for each activity: 1) the time spent by health personnel in IEC design, pretesting, and communication materials production; 2) number and frequency of allowances paid to health personnel during these activities; 3) number and frequency of supplies needed for the activities; 4) equipment used; and 5) other costs.

Figure 9. Screenshot of Communication Materials Worksheet

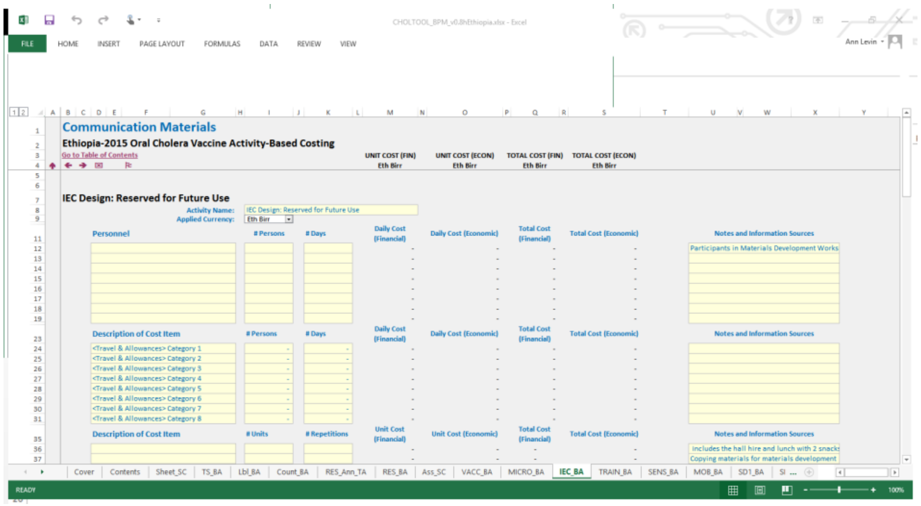

D. Estimate Training Activity Costs

In the training worksheets (see Figure 10), the user should enter assumptions for estimating the cost of training activities – training of trainers, training of vaccinators, and training of vaccinators. For each of these, fill in the quantities of resources used to conduct the trainings:

1) The time spent by health personnel or facilitators to conduct the trainings on conducting OCV campaigns;
2) The number and frequency of allowances paid to trainees during trainings;
3) The number and frequency of supplies needed for trainings
4) Equipment used in trainings;
5) Other costs used in trainings.

Figure 10. Screenshot of Training Worksheet

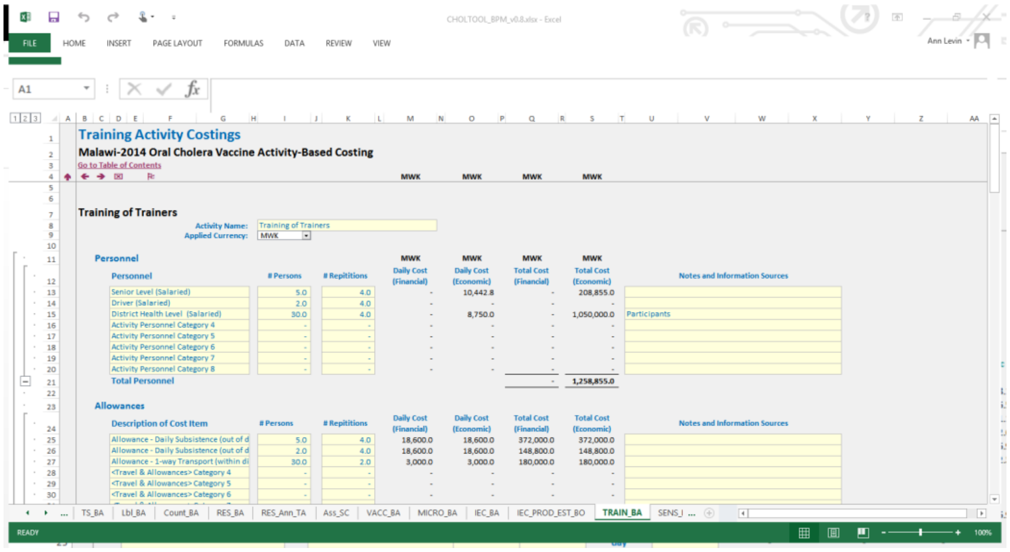

E. Estimate Pre-Campaign Sensitization Activity Costs

In the worksheet on sensitization activities at the national level and other levels such as district and health facility catchment areas, the user should enter assumptions or actual use of resources to estimate the cost of sensitization activities. These would include the following:

1) The amount of time spent by health personnel to conduct sensitization activities
2) The number and frequency of allowances paid to health personnel during sensitization activities;
3) The number and frequency of supplies needed for sensitization activities;
4) Equipment used in sensitization activities; and
5) Other costs used in sensitization activities.

F. Estimate Social Mobilization Activity Costs

The worksheet on social mobilization has sections on Round 1, Round 2, and the Campaign Launch. Fill in assumptions on use of resources to estimate the cost of social mobilization activities. These would include the following:

1) The time spent by health personnel to conduct social mobilization activities;
2) The number and frequency of allowances paid to health personnel during social mobilization activities;
3) The number and frequency of supplies needed for social mobilization activities;
4) Equipment used in social mobilization activities; and
5) Other costs used in social mobilization activities.

G. Estimate Vaccination Site Deployment Costs

In this worksheet (see Figure 11), the user should enter assumptions for estimating the cost of administering (giving) OCV vaccinations to members of the target population. These include the following:

1) The type, number, and cost of health personnel who conduct each vaccination actvity;
2) The number and frequency of allowances paid to health personnel for vaccination activities;
3) The number and frequency of supplies needed for vaccination;
4) Equipment used in vaccination activities; and
5) Other costs used in vaccination activities.

Figure 11. Screenshot of Vaccination Site Costing Worksheet

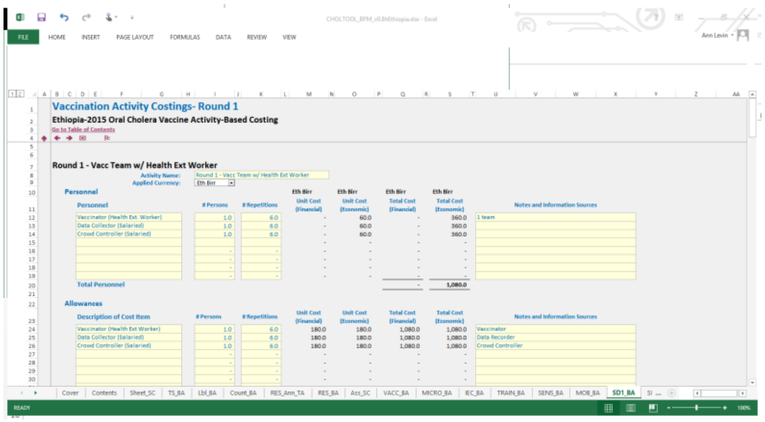

STEP 3: ESTIMATE NUMBERS OF EACH ACTIVITY

The next step is to 'ESTIMATE NUMBERS OF EACH ACTIVITY.' There are eight worksheets and these present program outputs by activity. The output worksheets include the following:

1) Vaccine procurement and delivery;
2) Microplanning;
3) Sensitization;
4) Communications material;
5) Training;
6) Social mobilization;
7) Vaccination activity costing – round 1; and
8) Vaccination activity costing – round 2.

A. Estimate the Number of Each Vaccine Procurement and Delivery Activity Needed

This worksheet shows the costs of vaccines by financial and economic costs. The costs are shown in two currencies – local and US$ or EURO.

B. Estimate the Number of Each Microplanning Activity Needed

In this worksheet (Figure 12), fill in the number of activities at each level. The second worksheet shows the disaggregated cost of micro-planning activities and the total cost.

Figure 12. Screenshot of Microplanning Worksheet

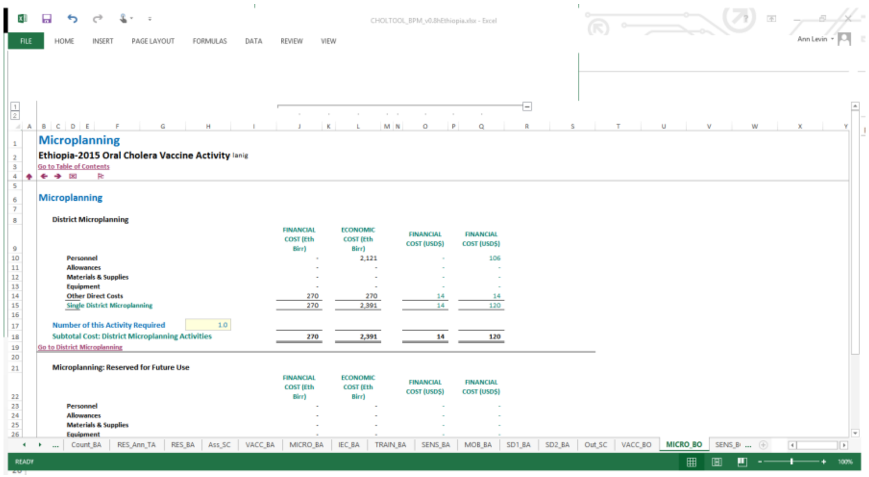

C. Estimate the Number of Each Sensitization Activity Needed

In this worksheet, fill in the number of sensitization events that will take place or have taken place in the yellow boxes. These will then be multiplied by the cost per activity to get total costs.

D. Estimate the Number of Each Sensitization Activity Needed

In this worksheet, fill in the number of communication material activities that will take place or have taken place in the yellow boxes.

E. Estimate the Number of Each Training Activity Needed

In this worksheet, fill in the number of training activities that will take place or have taken place in the yellow boxes.

F. Estimate the Number of Each Social Mobilization Activity Needed

In this worksheet, fill in the number of social mobilization activities that will take place or have taken place in the yellow boxes. This includes activities such as drama performances and public announcements.

G. Estimate the Number of Each Activity Needed During Round 1 of Vaccination

In this worksheet, fill in the number of activities needed for Round 1: 1) vaccination rounds, 2) # of command HQ support events; and 3) mop-up in vaccination.

H. Estimate the Number of Each Activity Needed During Round 2 of Vaccination

In this worksheet, fill in the number of activities needed for Round 2: 1) vaccination rounds, 2) # of command HQ support events; and 3) mop-up in vaccination.

STEP 4: REVIEW RESULTS AND ANALYSIS

The fourth section shows the results and analysis of the cost analyses. The two worksheets are the following: 1) COST SUMMARY and 2) ANALYSIS.

A. Review the Cost Summary

In this worksheet, the summary of the costs by activity and by their total are presented. These are shown financial and economic costs as well as in local and international currency. In addition, the cost per dose administered, the cost per partially vaccinated person and the cost per fully immunized person is shown.

B. Review the Analysis

In this worksheet, the results are shown in various charts. Figure 13 shows some of the charts in this worksheet.

Figure 13. Screenshot of ANALYSIS Worksheet

Analysis

Oral Cholera Vaccine Activity-Based Costing

Go to Table of Contents

Infographic

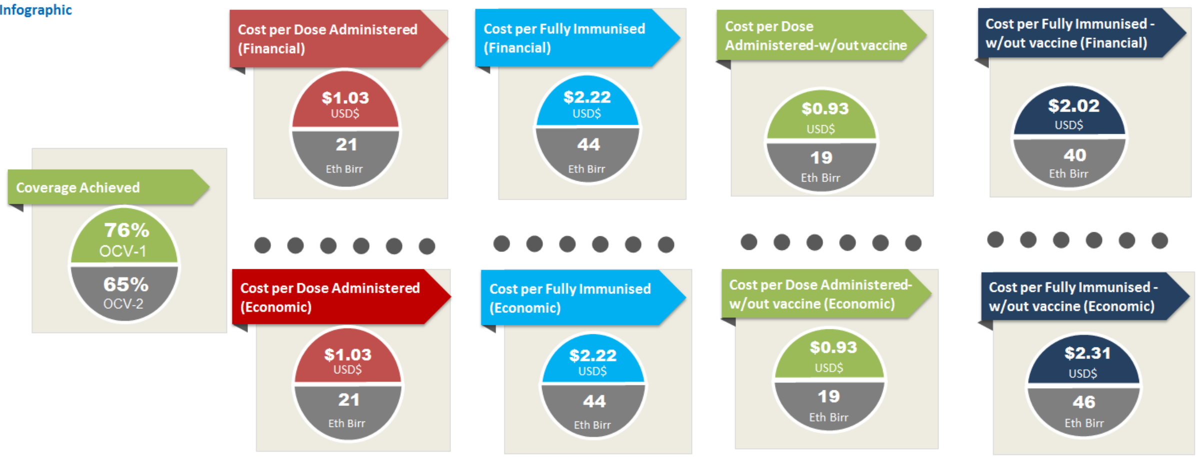

Tables

Activity Costs	FINANCIAL COST (Local Currency)	ECONOMIC COST (Local Currency)	FINANCIAL COST (USD$)	ECONOMIC COST (USD$)
Vaccine Procurement and Shipment	8,156	8,156	$8,156	$8,156
Microplanning	270	2,391	$14	$120
Sensitisation	30,392	30,863	$1,520	$1,543
Communication Materials	33,600	134,307	$1,680	$6,680
Training	42,700	49,199	$2,135	$2,460
Social Mobilisation	74,020	75,198	$3,701	$3,760
Vaccination - Round 1	727,180	790,499	$36,359	$39,525
Vaccination - Round 2	727,180	790,499	$36,359	$39,525
**TOTAL**	**1,643,498**	**1,881,112**	**$ 89,923**	**$ 101,768**

Cost per Immunization	FINANCIAL COST (Local Currency)	ECONOMIC COST (Local Currency)	FINANCIAL COST (USD$)	ECONOMIC COST (USD$)
Cost per Vaccine Administered (including vaccine)	19	21	$1.02	$1.16
Cost per Vaccine Administered (without vaccine cost)	19	21	$0.93	$1.07
Cost per Partially Immunised Person	35	40	$1.91	$2.16
Cost per Fully Immunised Person (with vaccine)	40	46	$2.21	$2.50
Cost per Fully Immunised Person (without vaccine)	40	46	$2.01	$2.30

Presentation of Results

The results on costs of OCV vaccination campaigns can be presented in tables to show the detailed breakdown of the projected resource requirements for each cost component. The numbers can be presented by intervention or by total amounts for each cost component.

Figure 14. Screenshot of ANALYSIS Worksheet Results Table

Analysis

Oral Cholera Vaccine Activity-Based Costing

Go to Table of Contents

Results (Number Receiving Oral Cholera Vaccine)

Number Receiving OCV-1 Only (Retrospective)	47,142
Number Receiving OCV-1&OCV-2 (Retrospective	40,707
Total Doses Administered (Retrospective)	87,849

It is very helpful to present the results through visuals such as charts and figures as well as tables so that the reader/audience can understand which components are cost drivers – i.e. costs that comprise a large share and influence on total costs. Charts are particularly useful to demonstrate differences in the importance of cost components to total resource requirements. When compared side by side, it is easy to see which are larger than others and could be considered ‘cost drivers’. Cost drivers are the largest cost categories.

Figure 15. Screenshot of ANALYSIS Worksheet Charts

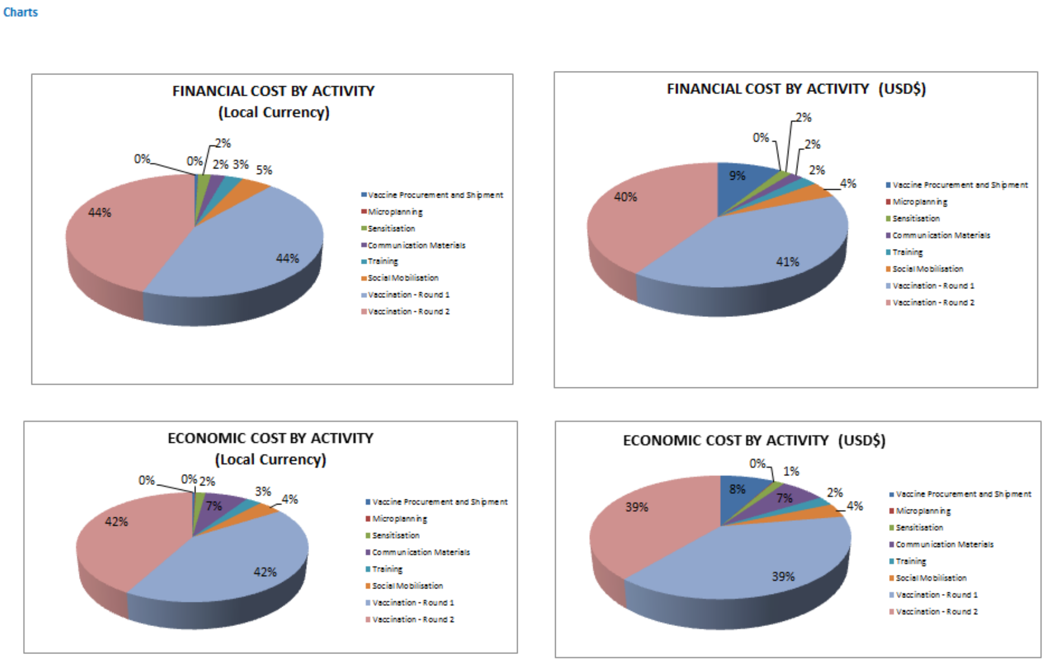

Results should be accompanied by a narrative explaining the findings in the tables, charts and figures.

The analyst should discuss the cost drivers for the OVC vaccination campaigns. It is often useful to present the total cost by activity in a pie chart or stack bar chart to show the relative proportions. It is also possible to compare the cost per dose administered by activity.

It is also useful to compare the costs by activity by Round 1 and Round 2 in pie charts or stacked bar charts to see if there are any differences in use of resources. Other possible comparisons are by vaccination sites or type of services delivery if sufficient information is available.

References

Siddique AK et al., 2009. El Tor cholera with severe disease: a new threat to Asia and beyond. Epidemiol. Infect.

Sur D, Dutta S, Sarkar BL, Manna B, Bhattacharya MK, Datta KK, Saha, Dutta B, Pazhani GP, Choudhuri AR, Bhattacharya SK. Occurrence, significance and molecular epidemiology of cholera outbreaks in West Bengal. Indian J Med Res 2007: 125: 772-776.

WHO, Weekly epidemiological record, March 2010. Cholera vaccines: WHO position paper. No. 13, 2010, 85, 117-128.
